# Regulation of p53 Stability and Apoptosis by a ROR Agonist

**DOI:** 10.1371/journal.pone.0034921

**Published:** 2012-04-11

**Authors:** Yongjun Wang, Laura A. Solt, Douglas J. Kojetin, Thomas P. Burris

**Affiliations:** The Scripps Research Institute, Jupiter, Florida, United States of America; University of Illinois at Chicago, United States of America

## Abstract

Activation of p53 function leading to cell-cycle arrest and/or apoptosis is a promising strategy for development of anti-cancer therapeutic agents. Here, we describe a novel mechanism for stabilization of p53 protein expression via activation of the orphan nuclear receptor, RORα. We demonstrate that treatment of cancer cells with a newly described synthetic ROR agonist, SR1078, leads to p53 stabilization and induction of apoptosis. These data suggest that synthetic ROR agonists may hold utility in the treatment of cancer.

## Introduction

In approximately 50% of human cancers the p53 gene is mutated, but in the remaining half of cancers activation of p53 function is considered to be a valuable strategy for development of anti-cancer therapeutics. p53 plays a critical role in limiting cell proliferation and inducing apoptosis in response to cellular stress/damage and abnormal function of p53 is associated with cancers [1]. p53 function is tightly regulated by modulation of protein stability. Under most conditions, p53 protein is undetectable primarily due to interaction of p53 with the E3 ubiquitin ligase MDM2 and succeeding proteosomal degradation. A number of compounds that inhibit the MDM2-p53 interaction or the subsequent steps toward proteosomal degradation are under evaluation for their anti-cancer activity.

Epidemiological data indicates that disruption of circadian rhythmicity is associated with development of cancer [2], [3], [4], [5]. Based on these data, the World Health Organization has classified shift-work associated with a disrupted circadian rhythm as a probable carcinogen [6]. Disruption of the circadian rhythm in rodents leads to increased tumor progresssion [7], [8], [9], [10] and disturbances in the expression of critical clock genes has been noted in several breast and liver cancer cell lines [11], [12], [13], [14].

The retinoic acid receptor-related orphan receptor α (RORα) is a member of the nuclear receptor superfamily that plays a critical role in regulation of the circadian clock. RORα expression oscillates in a circadian manner and plays an important role in modulation of expression of core clock components such as BMAL1, CLOCK and NPAS2 [15], [16], [17], [18], [19]. RORα expression is induced in response to a variety of cellular stresses [20], [21] and is downregulated in several breast, prostate, and ovarian cancer cell lines [21]. Additionally, RORα is expressed at very low levels in many cancers [21] suggesting that low RORα expression may be one mechanism underlying tumorigenesis. Based on these reports we focused on identification of pathways where RORα may regulate cell proliferation.

## Results

**Figure 1:**
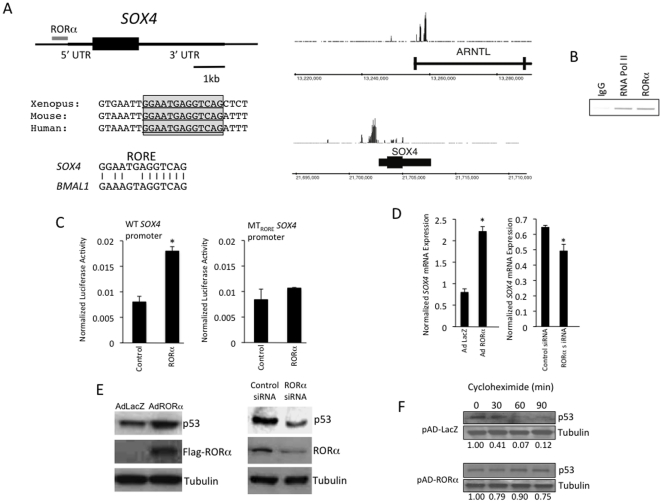
Identification of a RORE in the *SOX4* promoter. A) Schematic representing the *SOX4* gene. The single exon gene is shown in the diagram the untranslated regions are also shown. The area where significant RORα occupancy was detected in the ChIP-microarray screen is indicated above the gene. The putative RORE is shown below the gene structure and alignment illustrates absolute conservation between xenopus, mouse and human sequences. The RORE is indicated by the underlined sequence. Alignment of the putative *SOX4* RORE with the prototypic RORE from the prototypic ROR target gene, *BMAL1*. Right Panel: Screen shot from genome browser indicating regions generating signal from the ChIP/chip study on both *SOX4* and the positive control *ARNTL* (*BMAL1*). B) Chromatin immunoprecipitation assessing the occupancy of RORα at the *SOX4* promoter. IgG was used as a negative control and RNA polII was used as a positive control. C) Cotransfection assay where a luciferase reporter under the direction of the *SOX4* promoter was transfected into HEK 293 cells along with a vector directing the expression of RORα. Inclusion of RORα results in stimulation of luciferase expression. The second panel demonstrates that when the RORE is mutated in the *SOX4* promoter, which inhibits the ability of RORα to bind, RORα no longer has the ability to activate transcription of this reporter. WT, indicates wild type and MT, indicates mutant. Empty expression vector was included in the control wells. D) Adenoviral overexpression of RORα in HepG2 cells results in stimulation of *SOX4* mRNA expression relative to the LacZ adenovirus control. Suppression of expression of RORα using siRNA results in a reduction of *SOX4* mRNA. *, indicates p<0.05. E) Western blot illustrating that overexpression of RORα results in increased p53 protein while suppression of RORα expression results in decreased p53 protein expression. F) Analysis of the effect of RORα overexpression on the half-life of p53. HEK293 cells overexpressing either LacZ (control) or RORα were treated with cycloheximide for various amounts of time (0, 10, 60, 90 min) and p53 protein was assessed by western analysis and normalized to tubulin expression. Densitometry was used to assess expression and was signal was normalized to tubulin and the normalized relative (to time 0) expression is indicated below the blots. p53 displayed a half-life of 22±6 min (mean±S.E.) in the absence of RORα and a half-life >90 min with RORα overexpressed.

**Figure 2:**
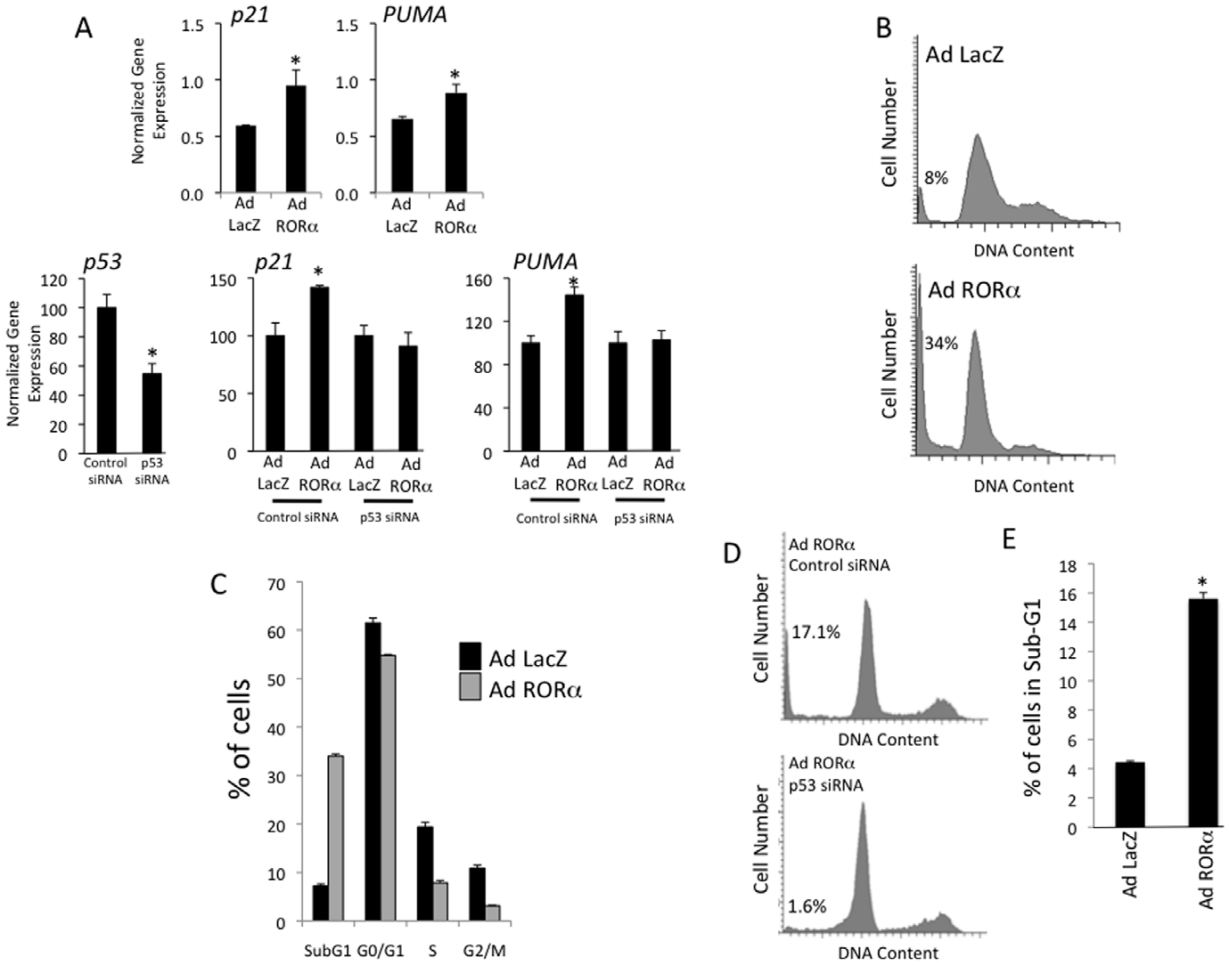
RORα regulates p53 function. A.) Overexpression of RORα in HepG2 cells results in increased expression of p53 target genes including *p21*, *BAX*, and *PUMA*. In control cells where the RORα adenovirus was not included, a control LacZ adenovirus was used. *, indicates p<0.05. Experiments shown in the lower panels were performed identical to the upper panels with the exception of inclusion of siRNA treatments as indicated. B) Cell cycle analysis of control HepG2 cells infected with LacZ adenovirus (top) or RORα adenovirus (bottom). C) Analysis of the number of cells in various stages of the cell cycle in control or RORα overexpressing HepG2 cells. D) Cell cycle analysis of HepG2 cells overexpression RORα (adenovirus treatment) after treatment with either control siRNA or p53 siRNA. E) Analysis of the number of MCF-7 breast cancer cells in sub-G1 infected with control adenovirus (LacZ) or RORα adenovirus. *, indicates p<0.05.

A chromatin immunoprecipitation (ChIP) – microarray screen was performed in the hepatocellular carcinoma cell line, HepG2, to identify RORα occupancy sites within the genome as we previously described [19]. We discovered RORα occupancy within the proximal promoter of the *SOX4* gene (Fig. 1A), which we found particularly intriguing because of its role in the regulation of p53 stability and function [22]. The tumor suppressor p53 plays a critical role in limiting cell proliferation and inducing apoptosis in response to cellular stress/damage and abnormal function of p53 is associated with cancers [1]. SOX4 directly interacts with p53 limiting its ability to be ubiquitinated by MDM2 and thus increases its stability [22]. In fact, induction of SOX4 expression is required for p53 stabilization in response to DNA damage [22]. Bioinformatic analysis of the RORα occupancy site revealed a putative ROR response element that was conserved between humans, mice and xenopus (Fig. 1A). We confirmed occupancy of the SOX4 promoter by RORα using a ChIP assay as shown in Fig. 1B. The *SOX4* promoter conveyed RORα-dependent regulation of a luciferase reporter gene in HEK293 cells as illustrated in Fig. 1C. This regulation was dependent on the RORE identified and shown in Fig. 1A since mutation of the RORE sequence rendered the construct unresponsive to RORα (Fig. 1D). Adenoviral overexpression of RORα in HepG2 cells resulted in an increase in *SOX4* mRNA expression whereas knock-down of RORα expression reduced *SOX4* mRNA expression in the same cell line (Fig. 1D). The limited effect on SOX4 mRNA after RORα knock-down may be due to compensatory actions of RORγ, which is known to act in concert with RORα in HepG2 cells [23]. Based on previous observations that altering SOX4 expression modulates p53 stability, we hypothesized that RORα expression may correlate with p53 protein stability [22]. Indeed, we observed that overexpression of RORα in HepG2 cells was associated with an increase in *SOX4* expression leading to increased p53 protein levels (Fig. 1D). Consistent with this observation, decreasing RORα expression in these cells leads to decreased p53 protein levels (Fig. 1E). We directly measured the stability of p53 under conditions where RORα was overexpressed by treating cells with cychoheximide and noted that overexpression of RORα clearly stabilized p53 expression (Fig. 1F). Additionally, overexpression of RORα led to increased expression of p53 target genes that play a key role in cell cycle arrest (*p21*) and apoptosis (*PUMA*) (Fig. 2A) [1]. Knock-down of p53 suppressed the ability of RORα overexpression to increase the expression of these genes (Fig. 2A lower panels). Further, analysis of HepG2 cells overexpressing RORα revealed that the number of cells in sub-G1 increased substantially over control cells (34% vs 8%) while cells in S and G2/M phase were also substantially reduced consistent with induction of apoptosis (Figs. 2B & 2C). These data are consistent with the observed increase in p53 stability and increase in *p21* and *PUMA* expression noted in Fig. 2B. Furthermore, the increase in sub-G1 cells induced by overexpression of RORα was blocked when p53 expression was knocked down demonstrating that RORα induction of apoptosis is p53-dependent (Fig. 2D). MCF-7 breast cancer cells show similar results when RORα is overexpressed; a significant increase in sub-G1 cells relative to control cells (15.5% vs 4.5%) (Fig. 2E).

**Figure 3:**
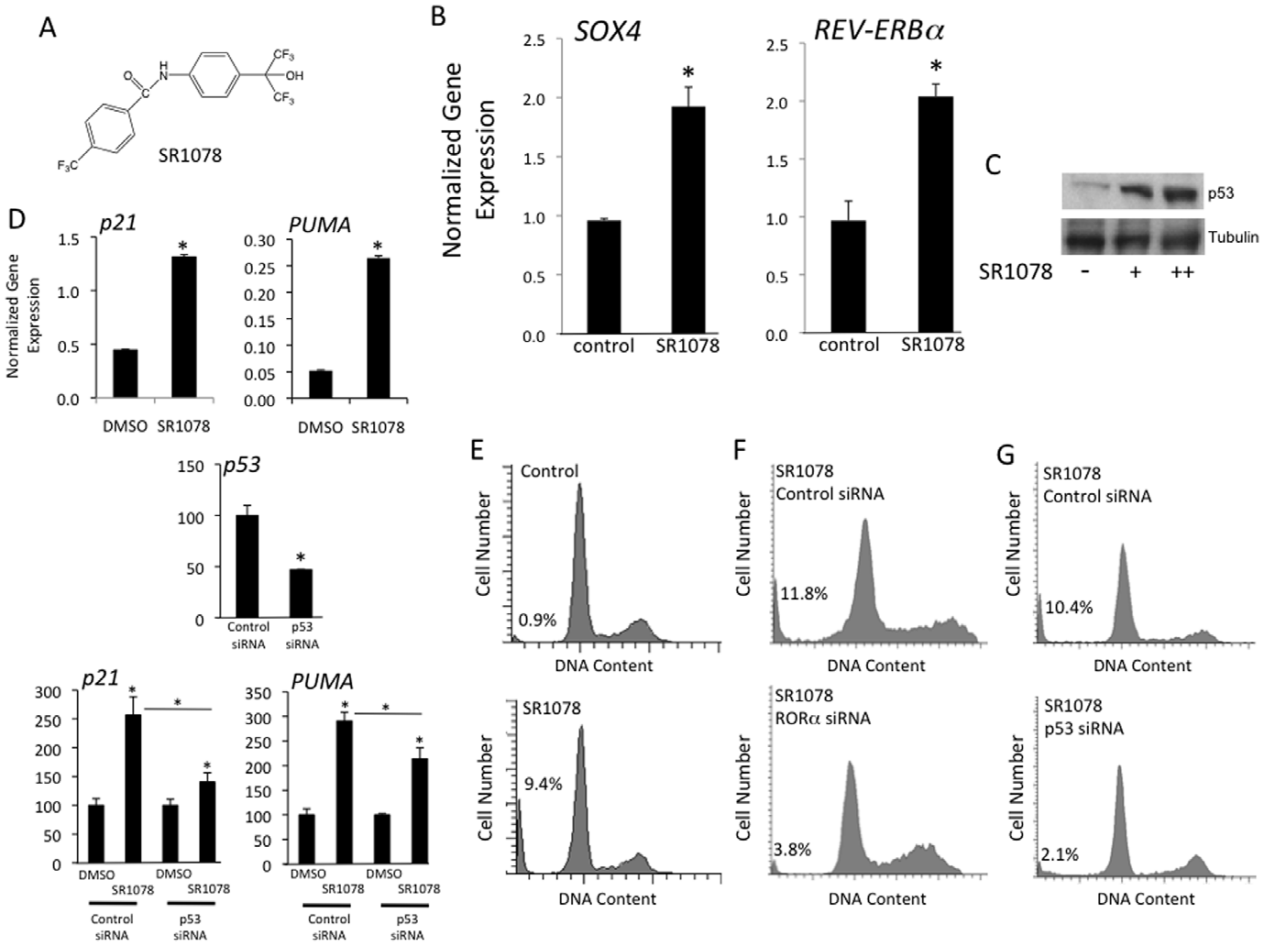
The RORα agonist, SR1078, increases SOX4 expression and p53 function. A) Chemical structure of SR1078. B) Treatment of HepG2 cells with SR1078 leads to an increase in *SOX4* and *REV-ERBα* mRNA expression. C) Treatment of HepG2 cells with SR1078 leads to increased p53 protein levels. + indicates 1 µM and ++ indicates 5 µM SR1078. D) Treatment of HepG2 cells with SR1078 leads to increased expression of p53 target genes, *p21* and *PUMA*. Experiments shown in the lower panels were performed identical to the upper panels with the exception of inclusion of siRNA treatments as indicated. E) Cell cycle analysis of control HepG2 cells treated with vehicle control or SR1078. Note the substantial increase in cells in sub-G_1_ following SR1078 treatment, 0.9% vs 9.4% indicated on the graph. F) Cell cycle analysis of HepG2 cells treated with either control siRNA or RORα siRNA in the presence of vehicle control or SR1078. G) Cell cycle analysis of HepG2 cells treated with either control siRNA or p53 siRNA in the presence of vehicle control or SR1078. *, indicates p<0.05.

Based on or results where overexpression of RORα leads to increased p53 protein stability, we examined the potential of a RORα agonist we recently identified to increase p53 stability. We recently characterized several synthetic ROR ligands including the first synthetic, selective ROR ligand, SR1078 (Fig. 3A) [24], [25], [26]. SR1078 functions as an agonist by activating RORα leading to an increase in transcription of RORα target genes [24]. Consistent with this activity, we noted that SR1078 induced the expression of *SOX4* mRNA in HepG2 cells as well as a well-characterized RORα target gene, REV-ERBα (Fig. 3B). We observe that SR1078 treatment also results in an increase in p53 protein levels (Fig. 3C) similar to the results we observed with overexpression of RORα. SR1078 treatment also led to a significant increase in the expression of p53 target genes *p21* and *PUMA* (Fig. 3D). Knock-down of p53 suppressed the ability of SR1078 to increase the expression of these genes (Fig. 3D lower panels). Consistent with the increase in p53 protein levels as well as the increase in the expression of p53 target genes, we found that SR1078 treatment led to increased apoptosis as indicated by the increase in HepG2 cells in sub-G_1_ (0.9% control vs. 9.4% SR1078) (Fig. 3E). The increase in apoptosis induced by SR1078 was both RORα- and p53-dependent since siRNA-mediated knock down of either of these genes suppressed the ability of SR1078 to increase cells in sub-G1 (Figs. 3F &3G).

## Discussion

The tumor suppressor protein p53 plays an essential role in regulation of key cellular processes including DNA repair, cell cycle, and apoptosis. In approximately half of all human cancers the p53 gene is deleted or mutated [1]. In many cancers with wild type p53, the activity of the tumor suppressor is inhibited by various effectors. One clear example of this is found in tumors where MDM2 is overexpressed due to an amplification of a chromosome segment that includes *MDM2* [27]. This leads to abnormal degradation of p53 and thus a similar phenotype to tumors with a mutant or deleted p53 gene. Inhibition of abnormal degradation of p53 is a logical pharmacological target and, in fact, several small molecule inhibitors of the MDM2-p53 interaction including nutlin-3, RITA, spirooxindoles and quilinols are being investigated as anti-cancer agents due to their abilities to increase cellular p53 protein levels through inhibition of MDM2-directed proteosomal degradation of p53 [28].

Our data suggests that a small molecule synthetic RORα agonist can increase p53 protein stability leading to increased p53 function and subsequent apoptosis. MDM2 inhibitors have been the focus of significant efforts to develop anti-cancer agents that function via activation of p53 activity. Here, we demonstrate that RORα agonists may also be useful for activation of p53 activity and thus represent a novel target for development of anti-cancer therapeutics. Most nuclear receptors that have identified ligands are well-characterized targets for drugs used in the clinic and the nature of the nuclear receptor ligand binding domain typically allows for optimization of small molecule ligands for drug development. Thus, RORα clearly represents a unique target for stabilization of p53 that is quite distinct from the challenging effort to inhibit a protein-protein interaction such as the MDM2-p53 interaction. While this manuscript was under revision, Kim et al. also described a role for p53 in regulation of p53 stability and function [29]. Although they show also that RORα regulates p53 stability they demonstrate a distinct mechanism from that proposed in this manuscript for increasing p53 stability involving the enhancement of p53-HAUSP interaction [29]. Kim et al indicate that they could not rule out additional non-HAUSP mechanism for regulation of p53 stability by RORα [29], which is consistent with our observation that RORα directly regulates the expression of *SOX4*, a critical gene involved in MDM2-dependent regulation of p53 stability.

## Methods

### Plasmids and viruses

The SOX4 promoter (−1121 to +90) was amplified from genomic DNA of HepG2 cells (ATCC, Manassas, VA) and cloned into pTAL-Luc luciferase report vector (Clontech, CA) to make the pTAL-SOX4 reporter construct. PGL4.73 reporter was from Promega (Madison, WI). pTrex-RORα and pTrex-RORγ were from Phenex Pharmaceuticals AG. RORα was tagged with FLAG and subcloned into pAd/CMV/V5-DEST vector through Gateway™ technique (Invitrogen). The adenovirus with FLAG-RORα was produced according to the manufacturer's instructions.

### Site-directed Mutagenesis

The SOX4 promoter mutant constructed by site directed mutagenesis as previously described [25], [30]. The RORE (−178 to −165) was mutated from GGAATGAGGTCAG to GGAATGAGGGGGG. The mutant primers targeting ROR binding site are: GCTCTGTAAATTGGAATGAGGGGGATTTGGAGCTTCTC (forward) and GAGAAGCTCCAAATCCCCCTCATTCCAATTTACAGAGC (reverse). The mutant primers were used to amplify mutant plasmid from pTAL-SOX4 reporter using *PfuUltra* HF DNA polymerase. The PCR product were treated with *Dpn* I to select for mutation-containing synthesized DNA and then transformed into XL1-Blue supercompetent cells. Positive clones were picked up and grew overnight in LB media. The plasmid were isolated using QIAprep Spin Miniprep Kit (Qiagen). The mutant construct was verified by sequencing.

### Cell culture and luciferase assay

HEK293 cells (ATCC) were maintained in Dulbecco's modified Eagle's medium (DMEM) supplemented with 10% fetal bovine serum (Invitrogen, Carlsbad, CA) at 37°C under 5% CO_2_. 24 h prior to transfection, HEK293 cells were plated in 96-well plates at a density of 15×10^3^ cells/well. Transfections were performed using Lipofectamine™ 2000 (Invitrogen). Each well was transfected with 20 ng pGL4.73, 50 ng ROR and 100 ng pTAL-Luc or mutant. Eight hours post-transfection, the cells were treated with vehicle or ligands. Twenty-four hours post-treatment, the luciferase activity was measured using the Dual-GloTM luciferase assay system (Promega). The value from experimental reporter was normalized to control reporter. The values indicated represent the means ± S.E. from four independently transfected wells. The experiments were repeated at least three times.

### Overexpression of RORα and siRNA Knockdown

The HepG2 cells (ATCC) were maintained in MEM supplemented with 10% fetal bovine serum at 37°C under 5% CO2. HepG2 cells were plated in 6-well plate one day before infection. The cells were infected with adenovirus for 24 hours and then switched to regular growth media. Twenty-four hours later, the cells were harvested to isolate total RNA. For knockdown assay, the control siRNA, human RORα siRNA, and human p53 siRNA (Thermo Scientific) were transfected with LipofectamineTM RNAiMAX (Invitrogen) by using reverse transfection. After 24 hours, cells were harvested to perform quantitative PCR assay or western blot.

### cDNA synthesis and quantitative PCR

Total RNA extraction and cDNA synthesis were performed as described before. The quantitative PCR was performed using ABI Prism 7900 HT detection system (Applied Biosystems, Foster City, CA). The primers for quantitative PCR are: human *RORα*, AAACAAGCAGCGGGAGGTGA (forward) and TGGCAAACTCCACCACATAC (reverse); human *SOX4*, GTGGTACAGGGGCAGTCAGT (forward) and AACACCATCACGATTCCGAT (reverse); Human *P21* TTAGCAGCGGAACAAGGAGT (forward) and CAACTACTCCCAGCCCCATA (reverse); human PUMA CTGTGCTCTGCCCGTGACCG (forward) and CTGGGGCGGCTTCAGCCAAA (reverse); human BAX GAGGATGATTGCCGCCGTGG (forward) and ACCCGGCCCCAGTTGAAGTT (reverse); human *CYPB*, GCAAATTCCATCGTGTAATCAAG (forward) and CGTAGATGCTCTTTCCTCCTG (reverse). The expression of target gene was normalized to housekeeping gene *CYPB*.

### Western Analysis

HepG2 cells were washed once with phosphate-buffered saline and then incubated for 10 min at 4°C in 100 µl of TNT lysis buffer (50 mM Tris-Cl, pH 7.5, 150 mM NaCl, and 1% Triton X-100) and a complete miniprotease inhibitor mixture (Roche Applied Science). Samples were then scraped and harvested into 1.5-ml microcentrifuge tubes, vortexed for 30 s, and then centrifuged (425× g for 10 min). Protein levels in the supernatants were determined using a Coomassie protein assay kit (Bio-Rad), and 20 µg of protein from each sample was separated by SDSPAGE (BioRad - 10%) and then transferred to a polyvinylidene difluoride membrane (Millipore, Milford, MA) and immunoblotted with primary antibodies: RORα (BioLegend), TP53 (Cell Signaling) or α-tubulin (Sigma) and horseradish peroxidase-conjugated secondary antibodies (Jackson Immunoresearch). Detection of the bound antibody by enhanced chemiluminescence was performed according to the manufacturer's instructions (Santa Cruz). For p53 half-life experiments, 10 µM cyclohexamide was added to the cells for 30, 60, or 90 minutes prior to harvesting the HepG2 cells for western analysis.

### FACS analysis

HepG2 cells and MCF7 cells (ATCC) were plated in 24-well plates the day before infection. The cells were infected with adenovirus 24 hours later. On the day of analysis, cells were harvested, washed, and fixed with 70% ethanolat −20°C. Fixed samples were washed with PBS twice and resuspended in propidium iodide staining solution for 30 min. Stained cells were analyzed on a FACScan flow cytometer (Beckton Dickinson).

### ChIP/chip screening

HepG2 cells were infected with adenovirus for 24 hours and then switched to regular growth media for another 24 h. The cells were harvested and sent to Genpathway for ChIP/chip assay as previously described [31], [32], [33]. The RORα ChIP/chip experiment has been previously described [19].

### Statistical Analysis

The Student's *t* test was used to test for significant differences between groups.
